# Effects of Orientation on Survival and Growth of Small Fragments of the Invasive, Clonal Plant *Alternanthera philoxeroides*

**DOI:** 10.1371/journal.pone.0013631

**Published:** 2010-10-26

**Authors:** Bi-Cheng Dong, Ming-Xiang Zhang, Peter Alpert, Guang-Chun Lei, Fei-Hai Yu

**Affiliations:** 1 College of Nature Conservation, Beijing Forestry University, Beijing, China; 2 Biology Department, University of Massachusetts, Amherst, Massachusetts, United States of America; Smithsonian's National Zoological Park, United States of America

## Abstract

**Background:**

The ability of small clonal fragments to establish and grow after disturbance is an important ecological advantage of clonal growth in plants and a major factor in the invasiveness of some introduced, clonal species. We hypothesized that orientation in the horizontal position (typical for stoloniferous plants) can increase the survival and growth of dispersed clonal fragments, and that this effect of orientation can be stronger when fragments are smaller and thus have fewer reserves to support initial growth.

**Methodology/Principal Findings:**

To test these hypotheses, we compared performance of single-node pieces of stolon fragments of *Alternanthera philoxeroides* planted at angles of 0, 45 or 90° away from the horizontal position, with either the distal or the proximal end of the fragment up and with either 1 or 3 cm of stolon left attached both distal and proximal to the ramet. As expected, survival and growth were greatest when fragments were positioned horizontally. Contrary to expectations, some of these effects of orientation were stronger when attached stolons were longer. Orientation had smaller effects than stolon length on the performance of fragments; survival of fragments was about 60% with shorter stolons and 90% with longer stolons.

**Conclusions/Significance:**

Results supported the hypothesis that orientation can affect establishment of small clonal fragments, suggested that effects of orientation can be stronger in larger rather than smaller fragments, and indicated that orientation may have less effect on establishment than amount of stored resources.

## Introduction

The ability of small clonal fragments to establish and grow after disturbance is an important ecological advantage of clonal growth in plants and a major factor in the invasiveness of some introduced, clonal species [1]–[4]. Natural disturbances such as floods, erosion and trampling often break off small units of one or a few ramets of clones and disperse them [2], [5], [6]. Ability of these fragments to establish probably contributes to the resilience of natural plant communities and may largely account for the large-scale spread of aquatic, introduced, clonal species. It is thus of scientific and practical interest to understand the factors that control the survival and growth of small clonal fragments.

One likely factor is the amount of stored resources contained in a fragment, and an important storage organ in some stoloniferous, clonal plants is the stolon [7], [8]. The internodes of stolons can store soluble carbohydrates and proteins [9], [10] that can be retranslocated to attached ramets [7], [11], [12]. Dong *et al*. (2010) recently showed that the survival and growth of ramets of *Alternanthera philoxeroides* increased with increasing length of attached stolons.

In this study, we build upon that previous finding to ask whether the orientation of dispersed, single-node clonal fragments can also affect their survival and growth, and whether this depends upon the length of attached stolon internodes, through their function as storage organs. After disturbance and dispersal, such small clonal fragments might become positioned in orientations away from the horizontal, which is the typical orientation for growth. Departure from the horizontal position may induce asymmetric distribution of endogenous growth substances (e.g. auxins) in the new buds of fragments, and make them reorient toward the vertical due to gravity stimulation [13], [14]. This could slow sprouting and increase reliance on stored resources in internodes. For instance, fragments of horizontal stems of giant reed (*Arundo donax*) had a higher sprouting ability than fragments of vertical stems when both types of fragments were positioned horizontally [15], [16], and this was related to differences in the distributions of auxins in the fragments. Horizontal orientation of fragments from the horizontal stems of clonal plants might thus increase establishment of the fragments and this effect might be stronger in fragments with shorter lengths of attached stolon internodes and thus less stored resources.

We therefore hypothesized 1) that horizontal orientation can increase the survival and growth of small clonal fragments, and 2) that this effect of orientation will be less in fragments with greater length of attached stolons. We tested these hypotheses in a greenhouse experiment with the invasive, stoloniferous plant *Alternanthera philoxeroides*.

## Results

**Figure 1:**
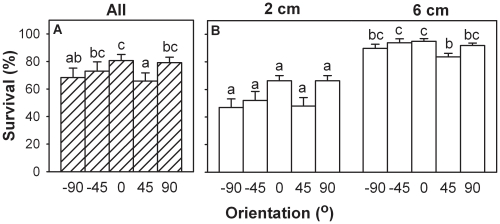
Effects of orientation and initial stolon length on survival of fragments of *Alternanthera philoxeroides.* Shaded bars (A) are grand means + s.e. of the five orientation treatments across the two stolon length treatments; open bars (B) are means + s.e. of the ten orientation and stolon length treatment combinations. Letters show which means differed within each measure (Student-Newman-Keuls test, *P* = 0.05).

**Figure 2:**
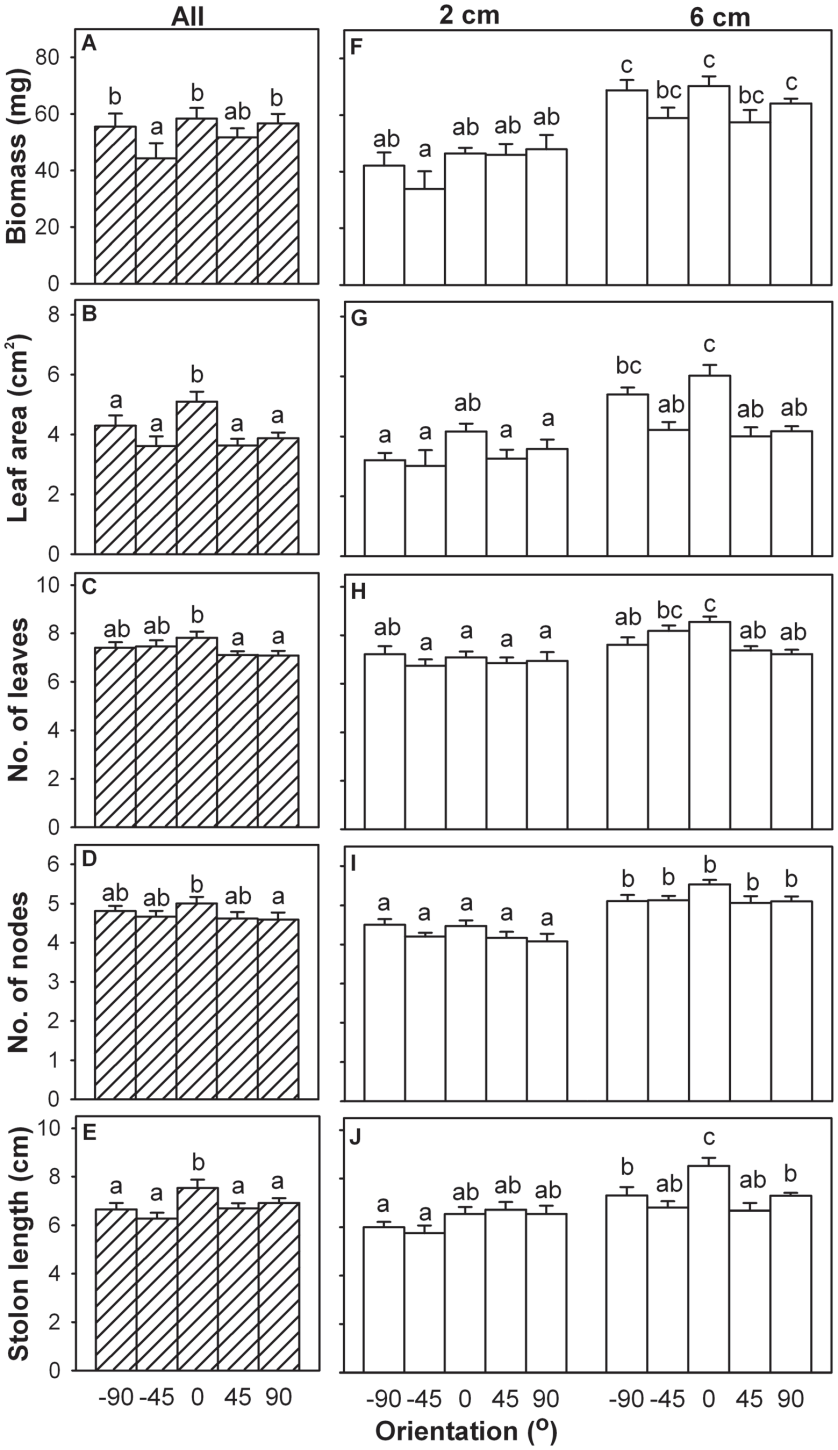
Effects of orientation and initial stolon length on final size of fragments of *Alternanthera philoxeroides.* Final size of fragments was measured by final dry biomass (A and F), leaf area (B and G), number of leaves (C and H), number of nodes (D and I), and total length of new stolons (E and J) of surviving fragments. Shaded bars (A–E) are grand means + s.e. of the five orientation treatments across the two stolon length treatments; open bars (F–J) are means + s.e. of the ten orientation and stolon length treatment combinations. Letters show which means differed within each measure (Student-Newman-Keuls test, *P* = 0.05).

The orientation of fragments had highly significant effects on their survival (Table 1) and final size (MANOVA: Wilk's λ = 0.36; *F* = 3.24; *P*<0.001). For each individual measure of size (Table 1), fragments performed the best in the 0° (horizontal) treatment (Figs. 1A and 2A–E). There was no evidence that performance of fragments planted at a given angle was better when their distal than when their proximal ends were oriented upwards (Figs. 1 and 2). Results thus largely met the prediction that survival and final size would be greatest when fragments were horizontal.

**Table 1:** ANOVAs for effects of orientation and stolon length on survival and measures of final size.

Effect	DF	Survival	Biomass	Leafarea	No. ofleaves	No. ofnodes	Stolonlength
Stolon length (S)	1,60	167.63***	63.81***	39.96***	24.88***	96.66***	29.75***
Orientation (O)	4,60	5.09**	2.46#	6.55***	2.70*	2.74*	5.02**
O×S	4,60	1.50^ns^	1.29^ns^	1.73^ns^	2.54*	0.76^ns^	3.19*

Values give *F*; symbols give *P*:

*** <0.001;

** <0.01;

* <0.05;

# <0.10;

ns ≥0.10.

The interactive effect of orientation and stolon length on final size of fragments was significant (MANOVA: Wilk's λ = 0.42, *F* = 2.68, *P*<0.001). In separate ANOVAs, effect of orientation interacted with effect of stolon length on two measures of size, number of leaves and length of new stolons (Table 1). Number of leaves, stolon length and leaf area did not differ among the five orientation treatments when the fragments were 2 cm long, but were the largest in the 0° (horizontal) treatment when the fragments were 6 cm long (Fig. 2G, H, J). Effects of orientation were thus greater when stolon length was longer, the opposite of the second predicted result.

Stolon length affected survival (Table 1) and final size of *A. philoxeroides* (MANOVA: Wilk's λ = 0.27, *F* = 28.30, *P*<0.001). By every measure, fragments performed better when attached to longer stolons (Table 1, Figs. 1 and 2). Length had a particularly strong effect on survival, which was about 60% overall in fragments with 2 cm of stolon attached at time of planting and 90% in fragments with 6 cm attached.

## Discussion

Survival and final size of single-node stolon fragments of *A. philoxeroides* were greatest when ramets were oriented in the horizontal position. This was consistent with the hypothesis that being positioned horizontally can increase the establishment of small clonal fragments such as might be created and dispersed by disturbance, and may provide the first published suggestion that tendency to lodge in the horizontal position could be advantageous in stoloniferous clonal plants.

Effects of orientation on establishment and initial growth of single-node clonal fragment of *A. philoxeroides* were not greater when nodes had shorter lengths of stolon internodes attached at time of planting, and were sometimes greater when nodes had longer attached stolons. These results were inconsistent with the second hypothesis. One possible explanation is that fragments actually have greater ability to respond to favorable conditions such as horizontal position when they have more stored resources. This could provide a revised hypothesis for further study.

Fragments with a greater length of stolon attached had a higher probability of survival, and achieved greater size if they survived. This was consistent with previous work on *A. philoxeroides* [4] and with studies on other clonal species [7], [17], [18], suggesting that positive effects of stolon internodes on survival of small clonal fragments are at least partly due to storage of reserves in stolons [7], [17], [18].

Given the relatively small effect of orientation observed in this study, it is uncertain whether the orientation of fragments following dispersal plays an important role in the ecology of *A. philoxeroides*, such as its ability to spread rapidly over large areas following introduction into a new region [19]–[21]. However, the study provides an initial indication that tendency of clonal fragments to become lodged in the horizontal position could increase their probability of establishment. Because length of stolons contributed greatly to the survival and growth of small clonal fragments, reducing the size of fragments and thus their storage material may be an effective method for the control of the invasiveness of *A. philoxeroides*.

## Materials and Methods

### Study species and propagation

*Alternanthera philoxeroides* (Mart.) Griseb., or alligator weed, is a perennial, stoloniferous herb in the Amaranthaceae. Native to the region of the Parana River in South America [19], [21], [22], *A. philoxeroides* has been introduced to and become highly invasive in many other regions, including southern China [20], [22], [23]. In China, *A. philoxeroides* rarely produces viable seeds [20] and instead reproduces asexually via stolons connected to established plants or through the establishment of dispersed fragments as small as a single ramet, i.e., a stolon node capable of bearing roots and two opposite leaves [24].

Dispersal of clonal fragments has resulted in a rapid expansion of the geographical range of *A. philoxeroides* in China [20], [25], where the species has become an important ecological and agricultural problem in both terrestrial and aquatic habitats [19], [26]. In water, *A. philoxeroides* can form dense mats and exclude native species [20]. On land, it invades pastures and agricultural fields as well as native plant communities [19], [20], [26].

Plants of *A. philoxeroides* were collected from Jiangxi Province in southeast China in mid-April 2009 and propagated vegetatively for 7 months in an artificial pond in Beijing. On 11 October 2009, 980 clonal fragments, each consisting of one unrooted stolon node with two leaves and the proximal and distal stolon internodes, were severed from the stock plants for use in the experiment. Both proximal and distal internodes of each fragment were at least 4 cm long and all fragments were derived from the mature, plagiotropic stems of the *A. philoxeroides* plants.

### Experimental design

**Figure 3:**
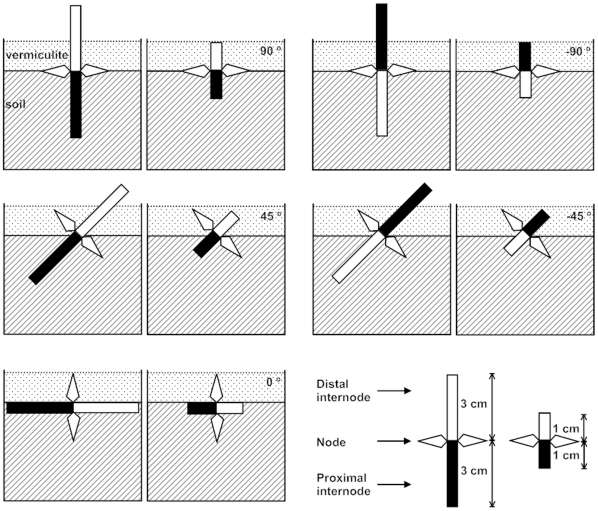
Experimental design. Single nodes of *Alternanthera philoxeroides* were oriented at −90, −45, 0, 45, or 90° to the horizontal position and left attached to a total of 2 or 6 cm of stolon.

The experiment used a factorial design with five orientation treatments (−90 −45, 0, 45, and 90°) and two stolon length treatments (2 and 6 cm). The orientation treatments combined departure from the horizontal position by 0, 45, or 90° with upwards (45 and 90°) or downwards (−45 and −90°) direction of the distal portion of the stolon (Fig. 3). We included the upwards and downwards variants to check whether fragments would perform better when the distal portion was upwards. For the stolon length treatments, either 1 or 3 cm of distal and proximal stolon were left attached (Fig. 3), for a total of 2 or 6 cm of attached stolon per fragment.

Fourteen fragments were randomly assigned to each of 70 plastic containers (37.7 cm long ×27.5 cm wide ×14.2 cm deep) filled with a 1∶1 mixture of sand and peat, to mimic a typical soil on which *A. philoxeroides* might be found on land in southern China. The node and leaves of each fragment were placed on the surface of the soil, which was then covered with a 1-cm-deep layer of vermiculite to help maintain soil moisture. To avoid potential photosynthesis by stolons and help isolate their effect on establishment of fragments via supply of stored resources, we wrapped the portions of stolons that were exposed to the light in tinfoil. Enough tap water was supplied to each container once a day to keep the soil moist. Seven containers were randomly assigned to each of the 10 treatment combinations; containers were treated as replicates.

The experiment started on 11 October 2009 and ended 9 weeks later on 11 December 2009. It was conducted in a heated greenhouse at Forest Science Co., Ltd., in Beijing Forestry University. The mean temperature in the greenhouse during the experiment was 15.3°C, as measured hourly by two Hygrochron temperature loggers (iButton DS1923; Maxim Integrated Products, USA).

### Measurements and analyses

On 11–14 December 2009, we counted numbers of nodes and leaves on each fragment; measured leaf area (WinFOLIA Pro 2004a, Regent Instruments, Inc., Québec, Canada) and length of new stolons; divided each fragment into roots, leaves, and new stolons; dried the portions at 70°C for 48 h; and weighed them. We did not include the stolon internodes left attached to fragments at the time of planting, because these stolon portions would necessarily differ in length and mass between stolon length treatments.

For analyses, we first calculated the percent survival and the means for each of the measures of size of the surviving fragments in each container. To test effects of treatments on survival, we ran an ANOVA with orientation and initial stolon length as fixed effects. To test treatment effects on measures of growth, we ran a MANOVA with the same effects and with all of the five measures of growth as dependent variables. Separate ANOVAs of each measure, reported along with the MANOVA, were also conducted. Student-Newman-Keuls tests (with *P* = 0.05) were used to test for differences between each of the ten combinations of the orientation and stolon length treatments and between the five orientation treatments across stolon length treatments. Values of survival were transformed to the arcsine of the square root and values of leaf area to the square root before analysis to increase normality and reduce heterogeneity of variance. Analyses were conducted with SPSS 16.0 (SPSS, Chicago, IL, USA).

We predicted (1) that survival and final size of fragments of *A. philoxeroides* would be greater when the angle of departure from the horizontal position was less and, for a given angle, when the distal end of the fragment was pointed upwards rather than downwards. We also predicted (2) that effects of orientation would be less when the length of attached stolon was greater.
